# More than just a name? From magnetic to optical skyrmions and the topology of light

**DOI:** 10.1038/s41377-024-01708-7

**Published:** 2025-01-03

**Authors:** Jian Chen, Andrew Forbes, Cheng-Wei Qiu

**Affiliations:** 1 School of Optical-Electrical and Computer Engineering, University of Shanghai for Science and Technology, Shanghai, 200093 China; 2 Department of Electrical and Computer Engineering, National University of Singapore, Singapore, 117583 Singapore; 3 School of Physics, University of the Witwatersrand, Johannesburg, South Africa

**Keywords:** Micro-optics, Nanophotonics and plasmonics

## Abstract

Topology is usually perceived intrinsically immutable for a given object. We argue that optical topologies do not immediately enjoy such benefits. Using ‘optical skyrmions’ as an example, we show that they will exhibit varying textures and topological invariants (skyrmion numbers), depending on how to construct the skyrmion vector when projecting from real to parameter space. We demonstrate the fragility of optical skyrmions under a ubiquitous scenario--simple reflection off an optical mirror. Optical topology is not without benefit, but it must not be assumed.

## Abstract (web summary)

Optical skyrmions formed by different permutations of the Stokes parameters of a skyrmionic beam will exhibit varying textures.

## Graphical abstract

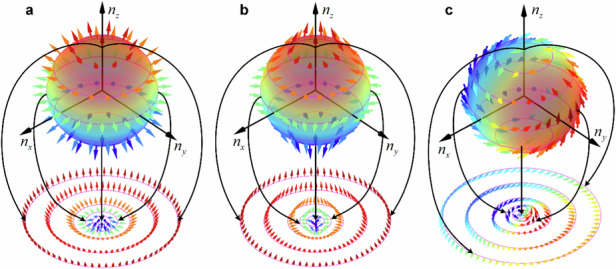

Skyrmions are field configurations with topological stability and particle-like characteristics that have been predicted and observed in a variety of areas, including twisted graphene^1^ Bose-Einstein condensates^2^, liquid crystals^3^, quantum Hall systems^4^, and notably in magnetic materials^5–11^. As illustrated in Fig. 1a, skyrmions have recently been extended to the optical domain^12^, triggering an emerging research trend with exponential growth (see Fig. 1b). Here the skyrmions are spin-textured fields in appearance only, where the polarization state is assigned a vector orientation for visualization. Although optical and magnetic skyrmions can be drawn in a visually similar manner, magnetic skyrmions are formed by electron spin in magnetic materials, with the Dzyaloshinskii-Moriya interactions (DMIs) playing a significant role in configuring the topological textures, where interaction and energy barriers preserve topology. In contrast, optical skyrmions have no natural energy barrier, and the components are independent in the absence of light-matter interaction. This raises important questions that have largely been ignored: are optical skyrmions more than just a name? From where might they derive their stability? Simply creating skyrmionic topologies and naming them does not imbue them with the same traits as their magnetic counterparts. The present exploration into optical skyrmions only unveils the tip of the iceberg, with the menacing questions sitting out of view below the surface. Here we bring these questions to the fore with some illustrative examples.

**Fig. 1:**
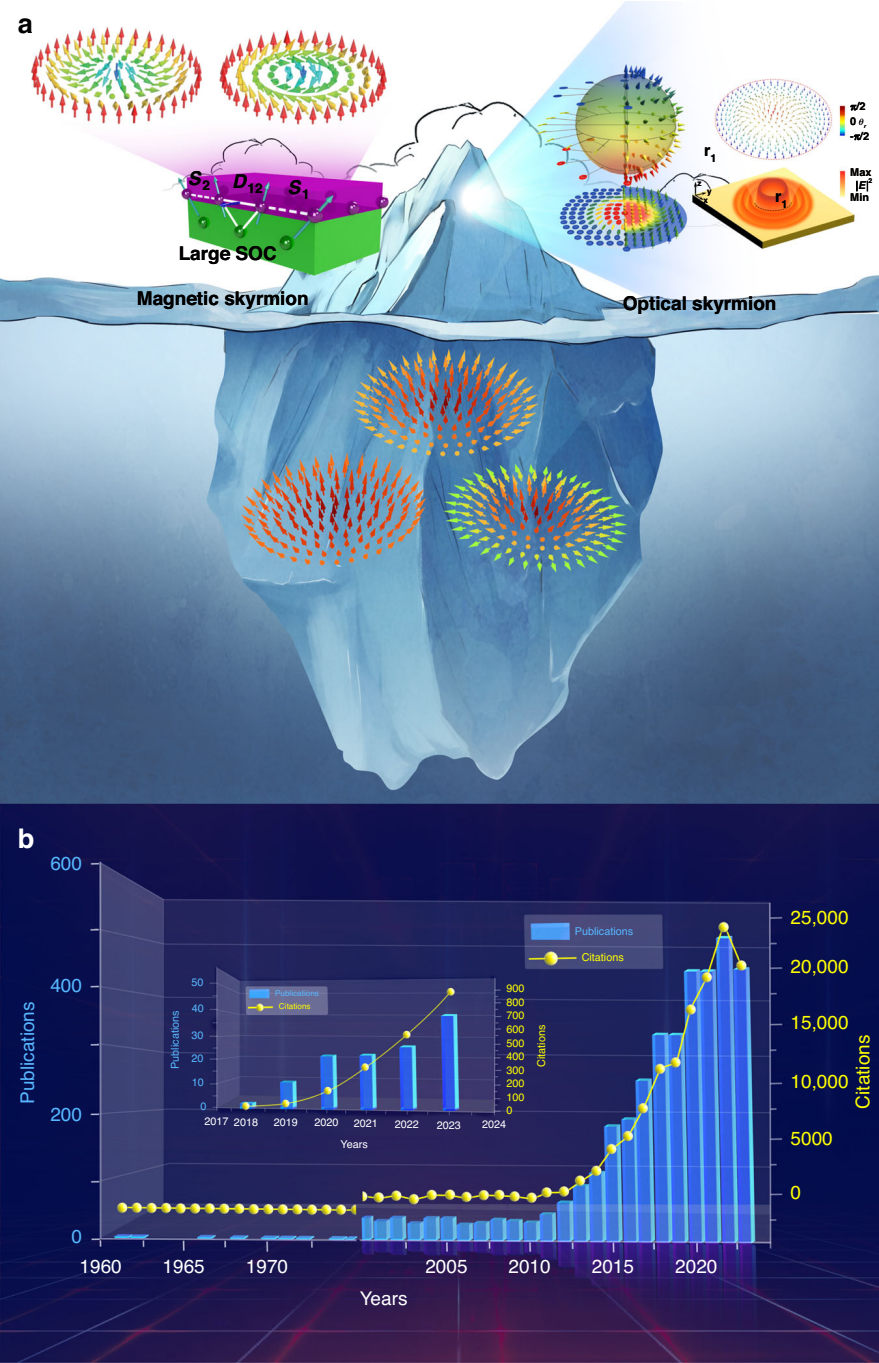
**Research status of skyrmions. a** Skyrmion iceberg. The current study of magnetic and optical skyrmions just unveils the tip of the iceberg, and numerous issues remain to be explored below the surface. For example, the non-integer skyrmion. **b** Publications and citations in the research of skyrmions. The blue histogram is the publications, and the yellow line and symbol present the citations. The main histogram and yellow curve give the statistical data of both magnetic and optical skyrmions, while the inset specifically shows the publications and citations in the study of emerging optical skyrmions. Figure reproduced with permission from: (**a**), ref. ^8^, © 2013 NPG, ref. ^10^, © K. Everschor, Univ. of Köln, ref. ^14^, © 2019 NPG, ref. ^15^, © 2020 APS

Consider first the construction of spin-textured optical fields. Without a magnetic field vector as a reference direction, how should we construct a coordinate system? Indeed, the construction of a skyrmionic topology requires a mapping from the S^2^ of real space to the S^2^ of parameter space, the latter described by a generalized skyrmionic vector ${\boldsymbol{n}}=[{n}_{x},{n}_{y},{n}_{z}]$. For instance, the evanescent electric field^13^ of surface plasmon polaritons excited in a hexagonally shaped coupling slit can construct the skyrmion vector as ${\boldsymbol{n}}=[{E}_{x},{E}_{y},{E}_{z}]$. The spin angular momentum (SAM)^14^ in evanescent optical vortex or focused free-space propagating beam is employed to form the skyrmion vector as ${\boldsymbol{n}}=[{s}_{r},{s}_{\varphi },{s}_{z}]$, where ${s}_{r}$, ${s}_{\varphi }$, and ${s}_{z}$ are the radial, azimuthal, and z component of the SAM. More commonly, the polarization Stokes parameters^15–17^ ${S}_{1}$, ${S}_{2}$, and ${S}_{3}$ of the appropriately customized vector beam are adopted to establish the skyrmion vector as ${\boldsymbol{n}}=[{S}_{1},{S}_{2},{S}_{3}]$.

However, different from the magnetic skyrmions whose topology is determined by the DMI constrained electron spin, the permutations of the three vector components in the optical skyrmions will influence their texture and skyrmion number (SN). Without loss of generality, we take the Stokes skyrmions as an example to illustrate this issue. For instance, a skyrmionic beam could be represented as $|\varPhi \rangle =|0\rangle |V\rangle +|1\rangle |H\rangle$, where $|V\rangle$ and $|H\rangle$ denote the vertical and horizontal states of polarization (SOP), respectively; 0 and 1 are the orbital angular momentum (OAM) carried by each polarized component (in units of $\hslash$). We can construct the skyrmion vector ${\boldsymbol{n}}$ by permuting the Stokes parameters ${S}_{1}$, ${S}_{2}$, and ${S}_{3}$ of the above skyrmionic beam. There are six permutations of ${S}_{1}$, ${S}_{2}$, and ${S}_{3}$ in total, three of which are shown in Fig. 2, each with its own topology. For ${\boldsymbol{n}}=[{S}_{2},{S}_{3},{S}_{1}]$, the resulting topology in Fig. 2a is Néel type skyrmion with SN of -1. While for ${\boldsymbol{n}}=[{S}_{3},{S}_{2},{S}_{1}]$, the obtained topology in Fig. 2b is anti-Néel type skyrmion with SN of 1. For other permutations, the resulting topologies are bimerons with varied textures and SNs (1 or -1); as shown in Fig. 2c, ${\boldsymbol{n}}=[{S}_{1},{S}_{2},{S}_{3}]$ gives rise to a bimeron with SN of -1. Thus, for optical skyrmions, the permutation of the adopted three vector components will cause changes to both the SN and the texture of the topology configured by these three components. It can also be found that one SN could correspond to several distinct topological textures. On the other hand, for one specific type of skyrmion, the evolution of the vector orientation in the topology will affect the sign of its SN. For example, the vector orientations in the Néel type skyrmion shown in Fig. 2a vary from downward to upward as they transition from the center to the outer periphery, thus, the corresponding SN is -1. To make the vector orientations vary from upward to downward as they transition from the center to the outer periphery, the associated SN will be turned to +1, which can be achieved by changing the orthogonal polarization basis and their phase difference used in the construction of skyrmionic beam.

**Fig. 2:**
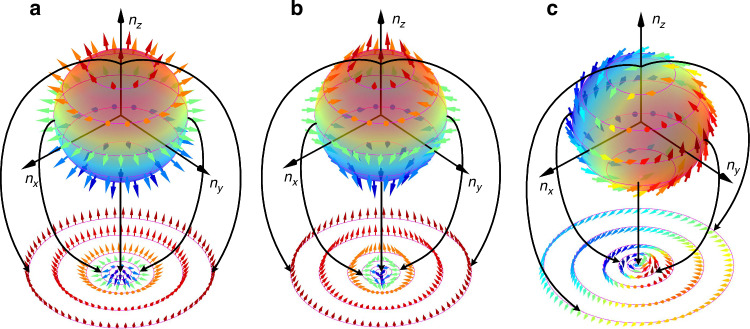
**Changeable textures**. Optical skyrmions are formed by different permutations of the Stokes parameters ${S}_{1}$, ${S}_{2}$, and ${S}_{3}$ of vector optical field $|0\rangle |V\rangle +|1\rangle |H\rangle$. The texture of each optical skyrmion can be obtained by mapping the vectors on a sphere onto a confined plane starting at the pole. **a-c**, Néel type skyrmion (**a**), anti-Néel type skyrmion (**b**), and bimeron (**c**) arise from the permutations $[{S}_{2},{S}_{3},{S}_{1}]$, $[{S}_{3},{S}_{2},{S}_{1}]$, and $[{S}_{1},{S}_{2},{S}_{3}]$, respectively. Both the sphere and the arrows are colored by the third component in each case

Now consider that we have decided on a nomenclature and vector choice so that the naming convention is at least consistent. In what follows we will adopt the skyrmion vector as ${\boldsymbol{n}}=[{S}_{2},{S}_{3},{S}_{1}]$. Does the optical topology inherit any of the stability properties of its magnetic counterpart? It might do, but in general it will not. Take the simple case of reflection off a mirror. When the skyrmionic beam $|0\rangle |V\rangle +|1\rangle |H\rangle$ is reflected by a mirror, the SOP of each component remains intact, while the chirality of the OAM carried by each component is flipped. Thus, the corresponding reflected beam is $|0\rangle |V\rangle +|\!-1\rangle |H\rangle$. The topological textures of the incident and reflected beams are shown in Fig. 3a. We note that the incident beam is a Néel type skyrmion, while the reflected beam is an anti-Néel type skyrmion, flipping sign in the topological invariant from +1 to −1, clearly an oxymoron. If we replace $|V\rangle$ and $|H\rangle$ with $|R\rangle$ and $|L\rangle$, then the incident and reflected skyrmionic beams can be expressed as $|0\rangle |R\rangle +|1\rangle |L\rangle$ and $|0\rangle |L\rangle +|\!-1\rangle |R\rangle$, respectively. As shown in Fig. 3b, both the topological textures and SNs are changed: topology is not conserved after the simplest of optical operations - reflection from a mirror. This simple example raises an important question about invariance: what parameter is conserved and to what type of perturbing system? The only experimental results^17^ on resilience have shown that (1) it is the coverage of the Poincare sphere that is invariant, and (2) it is invariant under any coordinate transformation of real space. Regarding (1): in general, this coverage can be non-integer, with the special case of mapping from spheres to spheres, which defines skyrmions, returning only integers. In this sense one must ask if the instance of skyrmionic mapping (integer maps) holds any special benefit? Regarding (2): not all optical transformations can be written as a coordinate transformation, and those that cannot will not immediately preserve skyrmionic topology. This has been demonstrated numerically for more complex media^18^, a warning sign that has been largely ignored in the emerging literature on topologies of light and their robustness: robustness must be tested and not assumed.

**Fig. 3:**
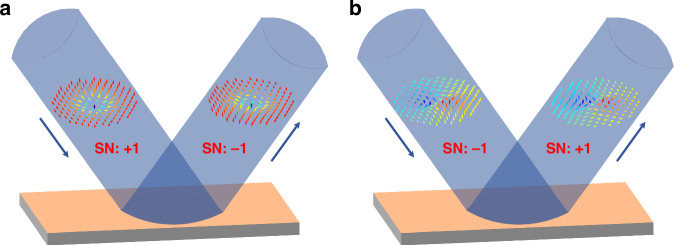
**Breaking skyrmionic topology by simple reflection. a** The incident skyrmionic beam is $|0\rangle |V\rangle +|1\rangle |H\rangle$ carrying Néel type skyrmion with SN of +1, while the reflected beam is changed to $|0\rangle |V\rangle +|\!-1\rangle |H\rangle$ possessing anti-Néel type skyrmion with SN of -1. **b** The incident skyrmionic beam is $|0\rangle |R\rangle +|1\rangle |L\rangle$ carrying bimeron with SN of -1, while the reflected beam is changed to $|0\rangle |L\rangle +|\!-1\rangle |R\rangle$ possessing another bimeron with SN of +1

Skyrmionic topologies of light hold tremendous potential as an exciting degree of freedom in structured light, offering a wide range of potential opportunities for optical information processing and transfer, data storage, high-resolution imaging and precision metrology to name but a few^12^. Here we have highlighted that optical skyrmions differ in a fundamental sense from their magnetic counterparts, demanding careful treatment of the nomenclature if the language of topology is to be consistent across disciplines, and careful thought to the physics of the systems under study before drawing conclusions on resilience. Optical topologies are rightly an exciting new avenue to explore, but let us do so with some care.
